# Computerization and the future of primary care: A survey of general practitioners in the UK

**DOI:** 10.1371/journal.pone.0207418

**Published:** 2018-12-12

**Authors:** Charlotte Blease, Michael H. Bernstein, Jens Gaab, Ted J. Kaptchuk, Joe Kossowsky, Kenneth D. Mandl, Roger B. Davis, Catherine M. DesRoches

**Affiliations:** 1 General Medicine and Primary Care, Beth Israel Deaconess Medical Center, Harvard Medical School, Boston, United States of America; 2 School of Psychology, University College Dublin, Dublin, Ireland; 3 School of Public Health, Brown University, Providence, Rhode Island, United States of America; 4 Division of Clinical Psychology and Psychotherapy, University of Basel, Basel, Switzerland; 5 Department of Anesthesiology, Critical Care & Pain Medicine, Boston Children’s Hospital, Harvard Medical School, Boston, United States of America; 6 Computational Health Informatics Program, Boston Children’s Hospital, Harvard Medical School, Boston, United States of America; 7 Department of Biomedical Informatics, Harvard Medical School, Boston, United States of America; 8 Department of Pediatrics, Harvard Medical School, Boston, United States of America; 9 Open Notes, Beth Israel Deaconess Medical Center/Harvard Medical School, Boston, United States of America; Medical University Graz, AUSTRIA

## Abstract

**Objective:**

To describe the opinions of British general practitioners regarding the potential of future technology to replace key tasks carried out in primary care.

**Design:**

Cross sectional online survey.

**Participants:**

1,474 registered GPs in the United Kingdom.

**Main outcome measures:**

Investigators measured GPs’ opinions about the likelihood that future technology will be able to fully replace–not merely aid–the average GP in performing six primary care tasks; in addition, if GPs considered replacement for a particular task likely, the survey measured opinions about how many years from now this technological capacity might emerge.

**Results:**

A total of 720 (49%) responded to the survey. Most GPs believed it unlikely that technology will ever be able to fully replace physicians when it comes to diagnosing patients (489, 68%), referring patients to other specialists (444, 61%), formulating personalized treatment plans (441, 61%), and delivering empathic care (680, 94%). GPs were not in agreement about prognostics: one in two participants (380, 53%) considered it likely that technology will be fully capable of replacing physicians in performing this task, nearly half (187, 49%) of whom believed that the technological capacity will arise in the next ten years. Against these findings, the majority of GPs (578, 80%) believed it likely that future technology will be able to fully replace humans to undertake documentation; among them 261 (79%) estimated that the technological wherewithal would emerge during the next ten years. In general, age and gender were not correlated with opinions; nor was reported burnout and job satisfaction or whether GPs worked full time or part time.

**Conclusions:**

The majority of UK GPs in this survey were skeptical about the potential for future technology to perform most primary care tasks as well as or better than humans. However, respondents were optimistic that in the near future technology would have the capacity to fully replace GPs’ in undertaking administrative duties related to patient documentation.

## Introduction

In 2013 economists Carl Frey and Michael Osborne at Oxford University’s Martin School estimated that in “in the next decade or two” more than half of all jobs would be substituted by intelligent technology [1]. In their forecast Frey and Osborne predicted that–at least in the short term–the work done by physicians and surgeons would be “low risk” (<1%) for automation, a prediction that is also shared by authors of a new working paper by the international Office of Economic Cooperation and Development [2]. According to some artificial intelligence (AI) experts, however, these predictions are too modest and machine level intelligence will be capable of replacing the basic functions of medical professionals by 2050 [3].

Within the medical community there has also been an upsurge in debate about the potential of big data and machine learning to disrupt healthcare professions [4,5]. In the UK ongoing pilots of chatbot apps covering 7.5 million people and aimed at triaging NHS care have attracted concerns about safety, raising questions about the testing of these devices and the regulation of the digital health industry [6,7]. Beyond the potential–or otherwise–of chatbots, opinions about the threat of AI appear to be divided. Some physicians continue to express skepticism arguing that, “concerns about physician ‘unemployment’ and ‘de-skilling’ are overblown” [8]. However, other physicians working in biomedical informatics and related fields predict that developments in AI are poised to revolutionize the delivery of health care [9,10,11,12], with some suggesting advancements may eventually obviate the need for physicians altogether [13].

Despite the abundant debate about the future of medicine, to our knowledge there is no exploration of the views of practicing physicians on the impact of AI on health professions. Given that much of the dialogue about machine learning technologies pivots on primary care, this survey seeks to determine the opinions of primary care physicians on the future of their job. We focus on currently registered general practitioners (GPs) working in the UK. Decomposing the work of primary care physicians into various foundational tasks (e.g. analyze patient information to reach diagnoses; provide empathic care, etc.) the survey asks UK GPs their views about whether, and when, future technology will be able to perform these tasks as well as or better than the average physician.

## Methods

### Study population

Participants in this cross-sectional national survey were randomly sampled from among UK-based general practitioners registered with the clinician marketing service Doctors.net.uk. This is the largest online medical network for medical doctors in the UK, providing information services to 228,317 physicians. In total, 87% of UK GPs are registered with the service [14]. Ethical approval for the study was obtained from Beth Israel Deaconess Medical Center, Harvard Medical School and University College Dublin.

We required 678 responses to reflect the population of registered UK GPs with 99% confidence (±5%). Based on previous surveys using Doctors.net.uk we predicted a response rate of around 46% [15,16,17]. Invitations to participate were emailed and displayed on the Doctors.net.uk homepages of 1474 randomly selected physicians during a period of one week, from 12^th^ June to 18^th^ June 2018. Our sample was stratified by gender and age using demographic information about currently registered UK GPs provided by the General Medical Council (GMC) [18]. All invited GPs were assured that their identities would not be disclosed to investigators, and participants gave informed consent before taking part in the survey. A small incentive of £10 ($13, €11) worth of ‘eSR’ points (exchangeable as shopping vouchers) was provided on completion, and participants were required to respond to every question to complete the survey. Further questions were embedded within the survey to determine whether respondents were currently practicing as GPs in the UK.

### Survey instrument

In light of the absence of research among physicians, the study team devised an original survey instrument to investigate the views of GPs about the potential impact of technology on the primary care profession (see S1 File). We developed the survey instrument in consultation with British and American general practitioners and piloted the survey with GP colleagues in the UK and USA (*n* = 12) to ensure face validity.

While the roles of primary care physicians vary from country to country, our aim was to formulate a generic list of common tasks performed in primary care with the ancillary goal that the survey might also be used for data collection beyond the UK. We used a validated primary care physician task list that encompasses 12 major professional functions and consulted with primary care physicians from the UK and USA to refine the list to six core tasks [19]. This list included: “analyze patient information to reach diagnoses”; “analyze patient information to reach prognoses”; “evaluate when to refer patients to other health professionals”; “formulate personalized treatment plans”; “provide empathic care to patients”; and “provide documentation (e.g., update medical records) about patients”. We aimed to ensure that the language in the task list was neutral and not biased in favor of either human physicians or technology in carrying out these functions.

In designing the survey instrument, we recognized the widespread acceptance among AI experts and informaticians that machine learning is likely to surpass humans in performing a number of core medical tasks, yet also noted some widespread vagueness in predictions about how, if at all, humans and machines might work together [20]. Many informaticians suggest that humans will be bypassed altogether for some tasks [9,10,13]; meanwhile, others argue that physicians will always be needed to manage patient care, and/or to undertake collaborative or ancillary responsibilities of key roles [8,21]. To avoid potential ambiguities in how respondents interpreted the questions and response options, we focused on whether tasks were likely to be fully rather than partially outsourced to technology. We also aimed to allow respondents to express discriminatory opinions about the tasks they considered most (or indeed least) vulnerable to replacement by machine learning. Finally, because the term “machine learning” may be unfamiliar among some physicians and considered too narrow a description among medical AI researchers, we employed generic language such as “machines” and “technology” to refer to AI innovations.

The first set of six items opened with a brief statement: “Some people believe that current and future innovations in artificial intelligence will lead to significant changes in medical practice and that machines will one day replace the work of physicians. Others deny that new technologies will ever have the capacity to replace this work”. We then asked respondents their opinion on the likelihood that “future technology will be able to fully replace and not merely aid human doctors in performing each task as well as or better than the average GP.” Employing 4-level Likert items we included the following response options: “extremely unlikely”, “unlikely”, “likely, and “extremely likely”. We avoided “don’t know”, “neutral” or “no opinion” options on the grounds that respondents often conflate these answers [22]. Furthermore, inclusion of these choices may have precluded measurement of substantive opinions among GPs: research indicates that this is a risk in self-administered questionnaires where time-pressured individuals may invest less effort in their answers [23]. Participants who responded that replacement was “likely” or “very likely” were asked a follow-up question about how soon in their estimation technology would have the capacity to perform the task and provided with a list of five response options.

A second set of items asked participants about their perceptions of the GP workforce. Using a five-point Likert scale we asked respondents for their opinions about whether the number of (i) GPs in the UK, and (ii) in their own practice, met patient demand. In the third section, respondents were surveyed on their current employment. We included two items about career satisfaction and burnout. To ensure that our survey was not over-long we employed Dolan et al.’s single-item measure of burnout that has been shown to be a reliable instrument for evaluating emotional exhaustion among primary care staff [24,25]. In this section we also asked participants about their work status (full time or part time); number of hours worked per week; and an estimate of the number of face-to-face patients that they saw each day. The purpose of these seven questions was to investigate whether physicians’ perceptions of the workforce, and/or current working conditions, were correlated with opinions about the future of medicine. A final section requested demographic information.

### Data management and analysis

We used descriptive statistics to examine physicians’ characteristics and opinions about the likelihood of future technology replacing GPs on six primary care tasks. In our analysis responses to each of the six tasks were collapsed into positive (for “likely” or “extremely likely” responses) versus negative (for “unlikely” or “extremely unlikely”) opinions. All correlation analyses were conducted using Spearman’s rho; all group comparisons were conducted using the Mann-Whitney U test. These analyses were chosen because, unlike Pearson’s *r* and *t*-tests, the dependent variable does not need to be continuous, and because most dependent variables in our analyses were ordinal outcomes. Alpha was set at 0.05 (two-tailed) for all analyses. All analyses were conducted using SPSS version 24.

## Results

### Respondent characteristics

Of the 1474 GPs who were invited to participate 720 responded (an overall response rate of 49%). There were more male (55%) respondents, the average year of qualification was 1996 (range 1963 to 2012, mode = 1998), and 51% were aged 45 or older. Most participants studied medicine in the UK (87%), with a sizeable minority describing themselves as of Asian, Black or minority ethnicity (30%). Respondents were from all regions of the UK. More of our respondents worked part time (54%). 83% of full-time participants reported working 41 hours or more each week while 78% of part time GPs reported working 21 hours or more. Among GPs who worked full time, the average number of patients seen per day was 34.7 (SD = 9.13) compared with 31.35 (*SD* = 9.41) for part time GPs (Table 1).

**Table 1:** Characteristics of the respondents and their practices*.

Characteristic	GPs (N = 720) *Number (percent)*
**Demographics**	
Male	397 (55.1)
Female	323 (44.9)
Age	
24–34	53 (7.4)
35–44	298 (41.4)
45–55	211 (29.3)
55–64	139 (19.3)
65 and above	19 (2.6)
Race/ethnicity	
Asian/Asian British	158 (21.9)
Black/African/Caribbean/Black British	9 (1.3)
Mixed/Multiple Ethnic Groups	14 (1.9)
White	500 (69.4)
Other Ethnic Group Not Listed	37 (5.1)
Studied medicine in the UK	624 (86.7)
**Employment**	
Full Time	331 (46.0)
Hours worked per week–no.	
Fewer than 10	21 (2.9)
10–20	71 (9.9)
21–30	115 (16.0)
31–40	173 (24.0)
41–50	206 (28.6)
More than 50	134 (18.6)
Patients seen per day–mean no. (SD)	33 (9.4)

*All the physicians who responded to the survey were registered GPs who were actively working in a primary care practice in the UK (see: S2 File).

Our participants varied from those registered with the General Medical Council (GMC) when it came to gender: 47% of UK general practitioners are male. However, respondents were of similar age to currently registered UK GPs where the mode year of qualification is 2000 and 55% are aged 45 and older. The GMC does not collect data on whether GPs work part time or full time.

### Opinions about technological replacement

Almost seven in ten participants (68%, 489/720) believed it is unlikely or extremely unlikely that technology will ever be able to fully replace human physicians when it comes to diagnostics (Table 2). Among those who believed replacement was likely or extremely likely approximately one third (35%, 82/231) estimated that the technological capacity would emerge in the next ten years (Table 3). When asked about referral of patients to other health professionals, 61% (444/720) considered it unlikely or extremely unlikely that technology would ever be able to perform this task as well as or better than the average GP; however, of the 39% who believed replacement was likely or extremely likely, 58% (160/276) estimated that the technological capability would arise in 0 to 10 years, including 15% (41/276) who gave an estimate of 0 to 4 years from now. Similarly, 61% (441/720) considered it unlikely or extremely unlikely that technology could ever fully replace humans when it comes to formulating personalized patient treatment plans; of the 39% who disagreed, around one in ten (12%, 34/279) gauged that the technological capacity will be available in up to four years from now with a further 44% (124/279) giving an estimate of between 5 to 10 years. Most GPs (94% 680/720) believed that technology would never be able to provide empathic care as well as or better than the average GP.

**Table 2:** Opinions about the likelihood of future technology replacing GP tasks*.

	Opinions *Frequency–percentage* *(95% CI)*			
Task	Extremely Unlikely	Unlikely	Likely	Extremely Likely
Diagnoses	24.2 (21.1 to 27.5)	43.8 (40.1 to 47.5)	28.3 (25.1 to 31.8)	3.8 (2.5 to 5.4)
Prognoses	13.5 (11.1 to 16.2)	33.8 (30.3 to 37.3)	45.8 (42.1 to 49.6)	6.9 (5.2 to 9.1)
Referral to other health professionals	15.3 (12.7 to 18.1)	46.4 (42.7 to 50.1)	33.6 (30.2 to 37.2)	4.7 (3.3 to 6.5)
Personalized treatment plans	21.9 (19.0 to 25.1)	39.3 (35.7 to 43.0)	35.1 (31.6 to 38.8)	3.6 (2.4 to 5.2)
Empathic care	69.0 (65.5 to 72.4)	25.4 (22.3 to 28.8)	4.9 (3.4 to 6.7)	0.7 (0.2 to 1.6)
Documentation	5.6 (4.0 to 7.5)	14.2 (11.7 to 16.9)	56.9 (53.3 to 60.6)	23.3 (20.3 to 26.6)

*All 720 respondents answered questions on the likelihood of technology replacing physicians on key tasks.

**Table 3:** Opinions about timescale for technological capacity to emerge*.

	Opinions *Frequency–percentage* *(95% CI)*				
Task *(Number)*	0–4 years	5–10 years	11–25 years	26–50 years	50+ years
Diagnoses *(n = 231)*	6.1 (3.4 to 10.0)	29.4 (23.6 to 35.8)	43.7 (37.2 to 50.4)	14.7 (10.4 to 20.0)	6.1 (3.4 to 10.0)
Prognoses *(n = 380)*	9.2 (6.5 to 12.6)	40.0 (35.0 to 45.1)	36.3 (31.5 to 41.4)	11.8 (8.8 to 15.5)	2.6 (1.3 to 4.8)
Referral to other health professionals *(n = 276)*	14.9 (10.9 to 19.6)	43.1 (37.2 to 49.2)	28.6 (23.4 to 34.3)	10.1 (6.8 to 14.3)	3.3 (1.3 to 4.8)
Personalized treatment plans *(n = 279)*	12.2 (8.6 to 16.6)	44.4 (38.5 to 50.5)	29.4 (24.1 to 35.1)	11.5 (8.0 to 15.8)	2.5 (1.0 to 5.1)
Empathic care *(n = 40)*	15.0 (5.7 to 29.8)	12.5 (4.2 to 26.8)	35.0 (20.6 to 51.7)	32.5 (18.6 to 49.1)	5.0 (0.6 to 16.9)
Documentation *(n = 578)*	34.1 (30.2 to 38.1)	45.2 (41.0 to 49.3)	16.4 (13.5 to 19.7)	3.5 (2.1 to 5.3)	0.9 (0.3 to 2.0)

*Only participants who believed technological replacement on a given task was “likely” or “extremely likely” were asked to provide an estimate of the timescale. Percentages refer to frequency of responses among those who responded to this question.

Against these findings, however, just over half of the participants surveyed (53%, 380/720) considered it likely or extremely likely that humans would be fully replaced when it comes to making prognoses; of these respondents around one in two (49%, 187/380) estimated that the technological capacity to do so would emerge during the next ten years.

The majority of GPs (80%, 578/720) also judged it likely or extremely likely that future technology will be able to replace human physicians when it comes to the task of documentation. Among those who answered affirmatively, approximately one third (34%, 197/578) believed the technological wherewithal would emerge in the next four years, with an additional 45% (261/578) giving an estimate of 5 to 10 years from now.

### Views about the GP workforce and employment

The overwhelming majority of participants (97%, 701/720) believed that the number of GPs in the UK is much less or somewhat less than patient demand. A similar number– 93% (667/720)–perceived the number of GPs in the community in which they practiced to be much less or somewhat less than patient demand. Nearly 4 in 10 GPs (38%, 272/720) reported that they were somewhat dissatisfied or very dissatisfied with their career, and 38% (269/720) reported burnout. These responses were similar to other reports on physician burnout in the UK [26]. There was a strong correlation between respondents’ level of reported satisfaction and burn out (ρ = 0.511, *p*<0.001). The number of patients seen by GPs per day was weakly correlated with level of reported satisfaction (ρ = 0.172, *p*<0.001) and burnout (ρ = 0.261, *p*<0.001).

### Correlates of opinions

In general, age and gender were not strongly correlated with opinions about technology replacing physicians. Only one task indicated a weak correlation, with more GPs in the younger age range (25–44 years old compared with 45 and older) believing that technology would replace physicians in formulating personalized treatment plans (ρ = 0.121, *p*<0.001, n = 720). A weak correlation between age and opinions about timescales was also found for diagnosis (ρ = -0.239, *p*<0.001, n = 231), prognosis (ρ = -0.148, *p* = 0.004, n = 380) and referral to other specialists (ρ = -0.167, *p* = 0.006, n = 276) with older GPs believing that the technological capacity would arise sooner. Gender of participants was weakly correlated with only one task–opinions about technology and the documentation of patient visits–with more male (82.6%) than female (77.4%) GPs believing physicians were likely to be replaced by technology (*p =* 0.013, n = 720). None of the following factors was associated with GPs’ opinions about technology and the future of primary care: reported burnout, level of job satisfaction, or whether GPs were full time or part time workers.

Participants who stated it is “extremely likely” that technology will replace the average GP in performing prognoses, making referrals, and in providing documentation estimated that technology would have this capacity sooner relative to those who said replacement was “likely” (*p<*0.001, n = 380; *p*<0.01, n = 276; *p*<0.001, n = 578, respectively).

## Discussion

### Summary of major findings

The opinions of physicians have been missing from the debate about the future of the medical professions. Most respondents to this survey (61–68%) considered it unlikely or extremely unlikely that technology will be able to fully replace humans in performing key primary care tasks including diagnosis, referral to other specialists, and the formulation of personalized treatment plans; though, as these figures reflect, a significant minority of participants disagreed with these opinions. GPs were evenly split over prognostics with just over half (53%) considering it likely or extremely likely that technology will be able to replace them in performing this task. Nearly one in two of these respondents estimated that the technological capacity will arise in the next decade. Taken together these finding indicate skepticism but also appreciable disagreement among UK-based GPs about the capacity of future AI to disrupt their profession.

Our data show that the overwhelming majority of UK GPs studied (94%) believed it was unlikely or extremely unlikely that technology will be able to provide empathic care to patients as well as or better than the average GP. In contrast with opinions about other tasks, most GPs were optimistic about the potential for technology to fully replace them in performing tasks related to patient documentation. Eight in ten expected that future technology will be able to undertake this role, and 79% of these respondents estimated that the wherewithal will emerge up to ten years from now.

The majority of physicians surveyed appeared to disagree with predictions that humans will be replaceable in performing key medical tasks; yet, interestingly, most respondents expressed confidence in the prospects for technology to replace them in undertaking paperwork. These opinions should also be put in the context of GPs’ perceptions about the current workforce: 97% of respondents believed that patient demand in the UK exceeds the number of available GPs, and nearly 4 in 10 (38%) reported burn out with similar numbers expressed dissatisfaction with their job. Many AI experts maintain that, in the long-term, technological advancements will lead to efficiencies in the delivery of care through progress in preventative medicine and diagnostic accuracy; better and cheaper access to health care; and reductions in physician workloads [27,28,29]. Against these claims, and despite perceptions about high patient demand and reports of professional burdens, our results suggest that respondents viewed future technology as likely to play a somewhat limited role in improving workflow efficiencies–as restricted to documentation. However, it is also conceivable that GPs may have multiple, possibly conflicting, beliefs about the impact of technology in primary care and research is needed to explore these issues further.

Finally, we found a weak correlation between age of GPs and estimates about timescales. Perhaps surprisingly, more older GPs (45 years and over) than younger GPs estimated that the technological capacity to replace physicians on the tasks of diagnosis, prognosis, and referral to other specialists, would arise sooner. In light of the strong recommendation from biomedical informaticians that medical schools urgently start to train physicians in computer science and related fields, our data provides evidence to inform ongoing debates about curricula reform as well as changes to pre-medical requirements [21].

Most experts in AI fields argue that medicine will be “revolutionized” by innovations in machine learning [4,9–11]. To the extent that some of these experts view core tasks as replaceable by machines, our findings suggest a disconnect between the opinions of practicing GPs’ and informaticians. Evidence of disparities in opinions between respondents and AI experts also warrants further exploration. More information is needed to compare the beliefs and attitudes of physicians working across different specialties with those of AI experts. In particular, it would be valuable to compare views on the potential benefits, limitations, and possible timelines for major advancements in machine learning in medicine. Last, but not least, future research should examine patients’ views and preferences when it comes to the role of technology in medical care [30].

### Strengths and limitations

This is the first survey to investigate physicians’ opinions about the future impact of technology on a medical specialty. While the majority of UK GPs are members of Doctors.net.org, a potential limitation of the study is that the participant response rate depended on individuals being logged into the network. The decision to complete the survey may have been influenced by the extent to which the responder was interested in the topic, and this, in turn, may have affected findings. The moderate response rate (49%) raises questions about representativeness, yet our respondents were reasonably similar to GPs registered with the GMC.

Some items on our list of tasks could be challenged on the grounds of vagueness. For example, it might be argued that the task “analyze patient information to reach diagnoses” does not encapsulate the subtasks or procedures involved in obtaining a diagnosis which might be said to include, among other things, efficient information gathering from the patient. However, to avoid building anthropocentric bias into our questions we deliberately avoided granular descriptions of GPs’ activities focusing on functions of primary care as opposed to how those functions might be realized.

While we obtained an optimal sample size for questions about the likelihood of technological replacement, this was reduced for items relating to time scales since only those participants who answered affirmatively were asked to respond to these questions. For some tasks this affected confidence intervals–e.g., empathy. Notwithstanding this limitation, since no other survey has been conducted on this issue, our investigation presents an important, foundational attempt to gauge physicians’ opinions on AI and future of a medical profession. In addition, the study investigated GPs’ opinions about whether technology will have the capacity to fully replace physicians on various tasks and not whether this will, in fact, happen. This latter, distinctive research question is also valuable and worth exploring. Finally, qualitative research might usefully probe physicians’ opinions about whether, and how, future innovations in technology might transform the designation of duties between humans and machines.

## Conclusions

The majority of GPs in this UK-wide survey were unconvinced about the potential for technology to perform as well as or better than humans when it comes to key primary care tasks, including diagnostics and referral to other specialists. Respondents were most skeptical about the future capacity for technology to replace GPs in the delivery of empathic care. In contrast, the majority of respondents were optimistic that, in the near future, AI would be fully capable of undertaking tasks related to paperwork (See Box 1).

**Box 1. Key questions and findings**

**What is already known about this topic**
- Experts working in biomedical informatics and related fields variously predict that advancements in artificial intelligence and machine learning will transform the medical professions and improve patient care.
- The potential for technology to overhaul clinical practice raises ethical and policy questions, including the preparedness of the medical profession to lead and adapt to change.

**What this study adds**
- Most UK GPs believed that future technology would not be able to perform as well as or better than the average GP in key primary care tasks including: diagnostics, referral to other specialists, and the provision of empathic care.
- In contrast, half of respondents believed that future technology would be capable of fully replacing humans in the task of prognostics; eight in ten believed this would be the case when it comes to paper work.
- Disparities in opinions exist between GPs and experts working in artificial intelligence, with GPs studied more skeptical about the role of technology in the future of the primary care.

Reviewing our findings, the skepticism observed among the majority of GPs in this survey might appear justified. Last year, following extensive publicity and high anticipation, the five-year collaboration between IBM Watson and the MD Anderson Cancer Center collapsed, suggesting that the future role of AI in healthcare may be more hype than reality [31]. Certainly, AI has yet to disrupt healthcare in ways that it has infiltrated other occupations and industries. However, as leading informaticians caution, cycles of hype camouflage broader historical trends: Amara’s Law is the observation that we tend to overestimate the effect of a technology in the short run and underestimate its effect in the long run [9,10]. We conclude by stressing that advancements in medical AI are unlikely to abate [28,29,32]. It is our hope that this survey provides prescient attention to the importance of the medical community leading, and fully engaging in, important but difficult debates about its future. Perhaps foremost among these questions is the adequacy of current medical education to inform future physicians about the scope as well as the current limitations of artificial intelligence, and data science [29]. Retooling, we argue, is necessary if the profession is to manage and deeply influence foreseeable change, including in the delivery of primary care.

## Supporting information

S1 File Survey questions. (DOCX)

S2 File Raw data. (XLSX)
