# Supplementary material for: Computerization and the future of primary care: A survey of general practitioners in the UK
Source: PLoS One. 2018 Dec 12;13(12):e0207418. doi: 10.1371/journal.pone.0207418 (PMC6291067; doi:10.1371/journal.pone.0207418)
Supplement: S1 File — (DOCX) [file pone.0207418.s001.docx]

**Supplementary File 1:**

**Survey Questions**

**Section I: Technology and the Future of Primary Care**

**The questions in this section are about your opinions on technology and the future of primary care.**

**Some people believe that current and future innovations in artificial intelligence will lead to significant changes in medical practice and that machines will one day replace the work of physicians. Others deny that new technologies will ever have the capacity to replace this work.**

**This survey focuses on your opinions about the future of general practice.**

**We are interested in your views on whether future technology will be able to perform various medical tasks as well as or better than the average GP.**

**In the questions below, we are interested in whether you believe technology will be able to FULLY REPLACE – NOT MERELY AID – GPs in performing these tasks.**

**The questions begin below.**

**In your opinion what is the likelihood that future technology will able to replace human doctors to perform these tasks as well as or better than the average GP?**

**1) Analyze patient information to reach diagnoses.**

[Options: Extremely unlikely; Unlikely; Likely; Extremely likely]

*If answered Likely or Extremely likely: Following question is asked:*

**(a) When, in your estimation, will technology have the capacity to replace the average GP in performing this task?**

[Options: 0-4 years; 5 to 10 years from now; 11 to 25 years from now; 26-50 years; more than 50 years from now]

**2) Analyze patient information to establish prognoses.**

[Options: Extremely unlikely; Unlikely; Likely; Extremely likely]

*If answered Likely or Extremely likely: Following question is asked:*

**(b) When, in your estimation, will technology have the capacity to replace the average GP in performing this task?**

[Options: 0-4 years; 5 to 10 years from now; 11 to 25 years from now; 26-50 years; more than 50 years from now]

**3) Evaluate when to refer patients to other health professionals.**

[Options: Extremely unlikely; Unlikely; Likely; Extremely likely]

*If answered Likely or Extremely likely: Following question is asked:*

**(c) When, in your estimation, will technology have the capacity to replace the average GP in performing this task?**

[Options: 0-4 years; 5 to 10 years from now; 11 to 25 years from now; 26-50 years; more than 50 years from now]

**4) Formulate personalized treatment plans for patients.**

[Options: Extremely unlikely; Unlikely; Likely; Extremely likely]

*If answered Likely or Extremely likely: Following question is asked:*

**(d) When, in your estimation, will technology have the capacity to replace the average GP in performing this task?**

[Options: 0-4 years; 5 to 10 years from now; 11 to 25 years from now; 26-50 years; more than 50 years from now]

**5) Provide empathetic care to patients.**

[Options: Extremely unlikely; Unlikely; Likely; Extremely likely]

*If answered Likely or Extremely likely: Following question is asked:*

**(e) When, in your estimation, will technology have the capacity to replace the average GP in performing this task?**

[Options: 0-4 years; 5 to 10 years from now; 11 to 25 years from now; 26-50 years; more than 50 years from now]

**6) Provide documentation (e.g., update medical records) about patients.**

[Options: Extremely unlikely; Unlikely; Likely; Extremely likely]

*If answered Likely or Extremely likely: Following question is asked:*

**(f) When, in your estimation, will technology have the capacity to replace the average GP in performing this task?**

[Options: 0-4 years; 5 to 10 years from now; 11 to 25 years from now; 26-50 years; more than 50 years from now]

**Section II: Perceptions of the Workforce**

**The questions in this section are about your perceptions of the GP workforce.**

For each of the following items, please indicate whether you think that overall…

**A. The number of GPs in the UK is…**

[Options include:

Much less than the demand; Somewhat less than the demand; About equal to the demand; Somewhat greater than the demand; Much greater than the demand]

**B. In the community in which you practice, the number of GPs is…**

[Options include:

Much less than the demand; Somewhat less than the demand; About equal to the demand; Somewhat greater than the demand; Much greater than the demand]

**Section III: Current Employment**

**The questions in this section are about your current employment.**

**A. Do you work full time or part time as a GP?**

[Options: Full Time or Part Time]

**B. In general, how satisfied are you with your career as a GP?**

[Options: Very satisfied; Somewhat satisfied; Somewhat dissatisfied; Very dissatisfied]

**C. Overall, based on your definition of burnout, how would you rate your level of burnout? In general, how satisfied are you with your career as a GP?**

[Options:

1 = I enjoy my work. I have no symptoms of burnout.

2 = Occasionally I am under stress, and I don’t always have as much energy as I once did, but I don’t feel burned out.

3 = I am definitely burning out and have one or more symptoms of burnout, such as physical and emotional exhaustion.

4 = The symptoms of burnout that I’m experiencing won’t go away. I think about frustration at work a lot.

5 = I feel completely burned out and often wonder if I can go on. I am at the point where I may need some changes or I may need to seek some help.

**D. How many hours do you work per week (including time spent seeing patients, home visits, administrative tasks, etc.)?**

[Options: Fewer than 10 hours; 10-20 hours; 20-30 hours; 30-40 hours; 40-50 hours; More than 50]

**E. On average how many patients do you see per day?**

Please enter number.

**Section IV: Physician Demographics**

**In this section we will ask demographic questions.**

**A. Gender. Please select.**

[Male or Female]

**B. Age. Please select.**

[Categories include:

25-34

35-44

45-54

55-64

65 and over]

**C. What year did you first start practicing medicine? Please select year.**

[Years listed as options]

**D. Where did you study medicine? Please select country.**

[List of countries provided as options]

**E. Race/ethnicity. Please select. [QUESTION IS OPTIONAL]**

[Categories to include:

Asian/Asian British

Black/African/Caribbean/Black British

Mixed/Multiple Ethnic Groups

White

Other ethnic group not listed]
